# Haploid Parthenogenetic Embryos Exhibit Unique Stress Response to pH, Osmotic and Oxidative Stress

**DOI:** 10.1007/s43032-023-01166-3

**Published:** 2023-01-23

**Authors:** Daphne Norma Crasta, Ramya Nair, Sandhya Kumari, Rahul Dutta, Satish Kumar Adiga, Yulian Zhao, Nagarajan Kannan, Guruprasad Kalthur

**Affiliations:** 1 Division of Reproductive Biology, Department of Reproductive Science, Kasturba Medical College, Manipal, Manipal Academy of Higher Education, Manipal, 576104 India; 2 Manipal Center for Biotherapeutic Research, Manipal Academy of Higher Education, Manipal, 576104 India; 3 Division of Clinical Embryology, Department of Reproductive Science, Kasturba Medical College, Manipal, Manipal Academy of Higher Education, Manipal, 576104 India; 4 Division of Reproductive Endocrinology and Infertility, Department of Obstetrics and Gynecology, Mayo Clinic, Rochester, MN USA; 5 Division of Clinical Core Laboratory Services, Department of Laboratory Medicine and Pathology, Mayo Clinic, Rochester, MN USA; 6 Division of Experimental Pathology and Laboratory Medicine, Department of Laboratory Medicine and Pathology, Mayo Clinic, Rochester, MN USA; 7 Center for Regenerative Biotherapeutics, Mayo Clinic, Rochester, MN USA; 8 Mayo Clinic Cancer Center, Mayo Clinic, Rochester, MN USA

**Keywords:** Haploid parthenogenetic embryos, Oxidative stress, Osmotic stress, pH stress, Endoplasmic reticulum stress, Mitochondrial membrane potential

## Abstract

Preimplantation-stage embryos are susceptible to various types of stress when cultured in vitro. Parthenogenetic embryos that lack spermatozoa contribution exhibit aberrant developmental dynamics due to their uniparental origin. Herein, we assessed whether the absence of paternal genome affects the susceptibility of the embryos to pH, osmotic and oxidative stress. Haploid parthenogenetic embryos (HPE) (activated oocytes with 1 pronucleus and 2 polar bodies) were generated by incubating cumulus oocyte complexes of Swiss albino mice with 10 mM strontium chloride for 3 h. Normally fertilized embryos (NFE) (fertilized oocytes with 2 pronuclei and 2 polar bodies) were derived using in vitro fertilization. At 2-cell stage, both HPE and NFE were exposed to various stressors including pH (6.8 to 8.2), osmotic (isotonic, hypotonic, and hypertonic), and peroxidatic oxidative (H_2_O_2_, 25 µM) stress. Endoplasmic reticulum stress response, mitochondrial membrane potential, and the rate of blastocyst development were assessed. HPE were susceptible to alteration in the pH that was well tolerated by NFE. Similarly, HPE displayed remarkable difference in sensitivity to hypertonic stress and oxidative stress compared to NFE. The results clearly indicate that the oocytes that develop into embryos in the absence of paternal contribution are more vulnerable to environmental stressors, further highlighting the importance of spermatozoa contribution and/or the ploidy status in mitigating these stressors and towards healthy early embryo development.

**Graphical Abstract:**

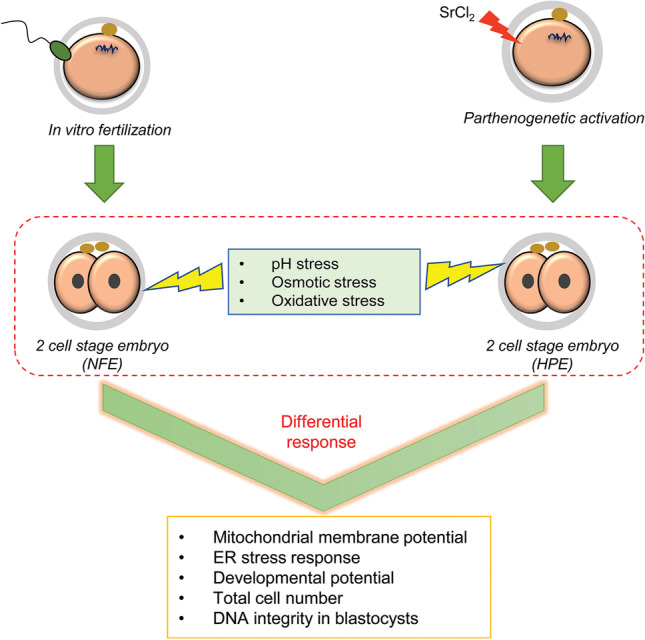

## Introduction

The developing embryos in vitro experience various types of stress due to the sub-optimal culture conditions [1]. These include fluctuations in temperature [2, 3], osmolarity [4], and pH [5], reactive oxygen species (ROS) [6, 7], or metabolic stress [8]. The preimplantation-stage embryos are highly vulnerable to these stressors, which are usually expressed as compromised developmental potential and poor embryo quality [9]. Stress may also directly impact gene expression patterns [8] and the synthesis, folding, transport, and post-translational modification of various proteins [10]. The preimplantation embryos exhibit essential adaptive responses to stressful conditions to improve their fitness and development [1, 11].

Early embryo development in mammals is thought to be mainly driven by maternally derived proteins [12] and organelles [13, 14]. The mitochondria undergo extensive changes in morphology and localization during preimplantation embryo development [15]. The mitochondrial membrane potential (MMP) controlled by the transport of H^+^ ions is absolutely crucial for ATP production [16]. Studies have shown that MMP at the 2-cell stage embryos influences their successful development [17, 18].

The initiation of the embryo genome activation (EGA), a key event in early embryo development, is greatly influenced by the maternally biased expressed genes (MBGs) [19]. Recent evidence suggests that spermatozoa also contribute to EGA, through miRNAs [20]. Failure in EGA can lead to embryo arrest or poor embryo development [21]. Maternal mRNA and proteins are found to regulate the DNA stability, transcriptional regulation, and protection against oxidative stress in a developing embryo until the zygotic genome activation occurs [22].

Cellular responses to most of the stressors converge at the endoplasmic reticulum (ER), with the induction of the unfolded protein response (UPR) and ER stress signaling (ERSS) response [10]. The UPR includes translational attenuation to stop the entry of new proteins into ER, transcriptional activation of genes encoding proteins that help to improve protein folding and in degradation of the misfolded proteins, and activation of apoptotic pathways to eliminate defective cells. This ultimately helps to re-establish the protein synthesis machinery [23]. Under challenging conditions, embryos express ER stress response proteins such as glucose-regulated protein 78 kDa (GRP78), a “master regulator” of the UPR [24] and X-box-binding protein 1 (XBP-1), the major downstream activator involved in the regulation of UPR expression [25]. Increased levels of endogenous GRP78 and XBP-1 in the in vitro cultured embryos are indicative of increased ER stress and are correlated with their poor embryo development [24, 25].

Several studies in the past have demonstrated that spermatozoa do not act as a mere transport means of the paternal genome into the oocyte, but also play a significant role in early embryo development [26, 27]. Transfer of microRNAs [26, 28], oocyte activation factors such as phospholipase C zeta [29] and post‐acrosomal WW‐domain binding *protein* (PAWP) [27, 30], and the centriole [31] have been reported by earlier studies. Further, the paternally biased expressed genes (PBGs) are known to regulate the determination of the first cleavage axis and late embryonic events such as compaction and trophectoderm specification [19]. An earlier study from our group has demonstrated that the absence of paternal factors alters the tolerance of embryos to ammonia during in vitro culture [32]. However, the role of paternal factors in regulating the susceptibility of preimplantation embryos to common culture environmental stressors such as pH, osmotic and oxidative stress remains unclear. The present investigation was carried out using normally fertilized embryos (NFE) and haploid parthenogenetic embryos (HPE) to assess the impact of the paternal factors on the oocyte fitness and embryo development in vitro upon exposure to common environmental stressors.

## Methods

### Animal Details

Inbred adult Swiss albino mice (6–8 weeks) maintained under standard conditions (23 ± 2 °C, 45–50% humidity, 12 h each of light and dark cycles, food, and water ad libitum at the Central Animal Research Facility, Manipal Academy of Higher Education, Manipal) were used for the experiments. The study was approved by the Institutional Animal Ethics committee of Kasturba Medical College, Manipal (IAEC/KMC/51/2015 and IAEC/KMC/56/2018).

### Superovulation and Oocyte Collection

The adult female Swiss albino mice were stimulated using 5 IU of pregnant mare serum gonadotropin (PMSG) and 10 IU of human chorionic gonadotropin (hCG) at an interval of 48 h. At 13.5 h post-hCG administration, the cumulus oocyte complexes (COCs) were collected by teasing the oviduct in M2 medium. The COCs collected were either subjected to in vitro fertilization (IVF) to obtain normally fertilized embryos (NFE) or to strontium chloride (SrCl_2_) activation to obtain haploid parthenogenetic embryos (HPE).

### In vitro Fertilization

**Fig. 1:**
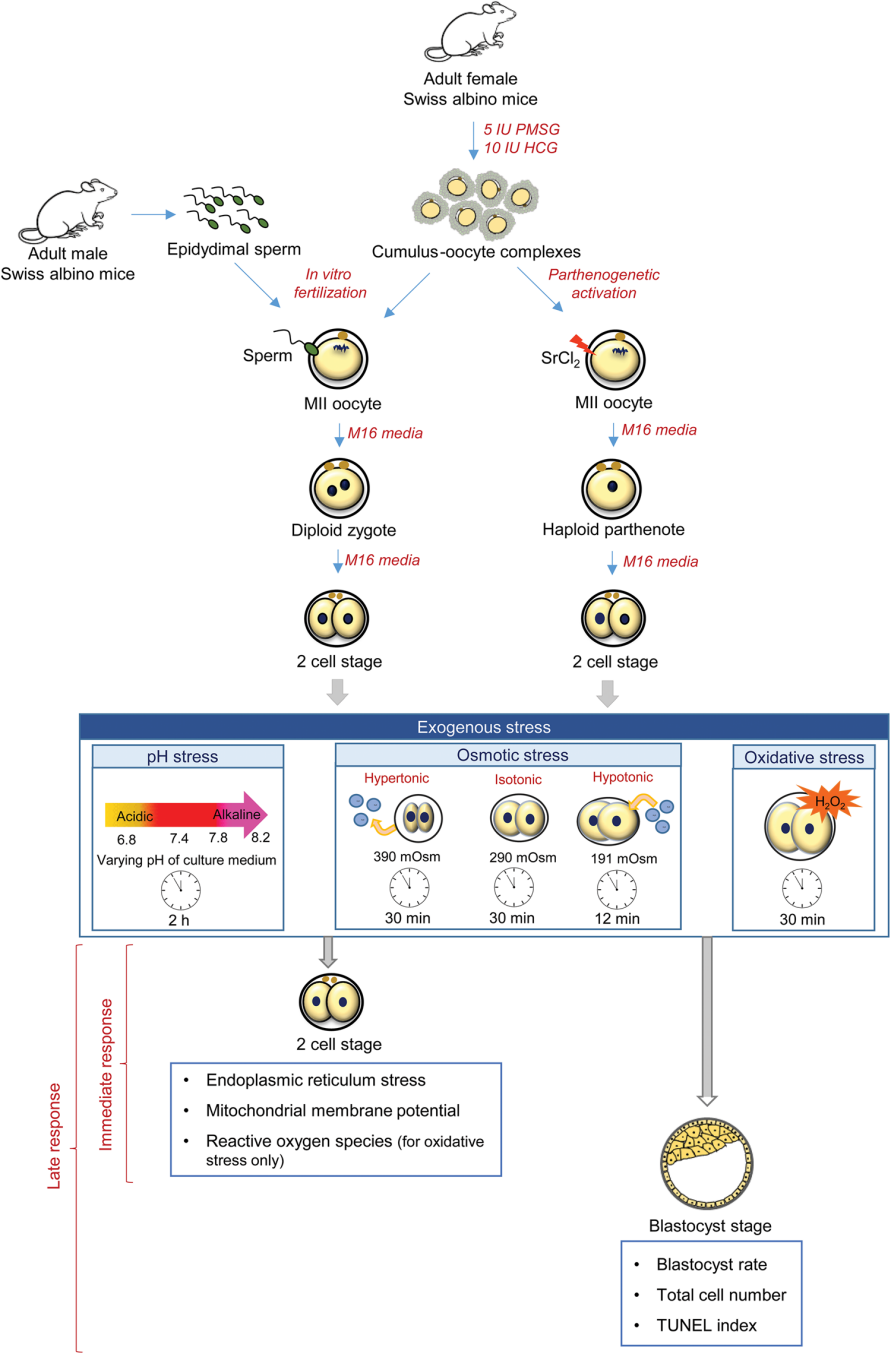
Schematic representation of the study outline showing generation of normally fertilized embryos and haploid parthenogenetic embryos, their exposure to various stress in vitro (pH, osmotic and oxidative stress), and the parameters used to assess the response in embryos

The cauda epididymis was collected from adult male Swiss albino mice. The caudal spermatozoa were released in prewarmed Earle’s balanced salt solution (EBSS) medium containing 0.1% bovine serum albumin (BSA) and incubated at 37 °C and 5% CO_2_ for 2 h to induce capacitation. The motile spermatozoa were collected by swim-up technique as described by Satish et al. [33]. Insemination droplets were prepared by placing 80 µL droplets covered with prewarmed mineral oil (Cat. No. 61822605001730, Merck Life Science Pvt. Ltd.). COCs were collected from superovulated female mice and randomly transferred to each insemination droplet incubated at 37 °C and 5% CO_2_. At 10–12 h post-insemination, the oocytes were denuded to remove cumulus cells, washed in M16 culture medium, and transferred to a culture dish containing M16 media droplets (20 µL) covered with prewarmed mineral oil. The fertilization was assessed under inverted microscope (Olympus IX71, Tokyo, Japan) by observing for 2 pronuclei and 2 polar bodies (2 PN/2 PB). The fertilized embryos were cultured in M16 media until they were used for further experiments (Fig. 1).

### Haploid Parthenogenetic Activation

The COCs were incubated in the activation medium (10 mM SrCl_2_ in Ca^2+^ and Mg^2+^ cations-free M16 media) for 3 h, after which the oocytes were denuded to strip the cumulus cells. The oocytes were washed and cultured in M16 media droplets (20 µL) covered with prewarmed mineral oil. Two hours post-activation, the oocytes were observed under the inverted microscope (400 ×) to identify the haploid parthenogenetic embryos (activated oocytes with 1 PN and 2 PB) [34].

### Exposure of NFE and HPE to M16 Media with Varying pH

NFE and HPE at 2-cell stage were randomly distributed and transferred to 20 µL drops of M16 medium (15 embryos per droplet) with different pH. M16 medium with pH 7.4 served as control medium. The pH of M16 media was adjusted to 6.8, 7.8, or 8.2 using 1 N HCl and 1 N NaOH. After incubation for 2 h under mineral oil at 37 °C and 5% CO_2_, the embryos were used for further experiments. Part of the embryos was used for assessing ER stress and MMP, while the remaining embryos were cultured in M16 media with pH 7.4 till the blastocyst stage.

### Exposure of NFE and HPE to Hypotonic and Hypertonic M16 Media

NFE and HPE at 2-cell stage were collected and randomly divided into three groups: (i) isotonic medium (M16 medium, 290 mOsmol/L); (ii) hypotonic medium (191 mOsmol/L; prepared by mixing M16 media with an equal volume of milliQ water); and (iii) hypertonic medium (390 mOsmol/L; prepared by dissolving 0.01 M sucrose in M16 media). The embryos were cultured in hypotonic media for 12 min and in hypertonic media for 30 min. The osmolarities of the culture medium for hypotonic and hypertonic stress were selected based on the earlier literature [35–37], and the duration of exposures was based on our preliminary experiments. ER stress and MMP were assessed in 2-cell stage embryos after exposure to different osmotic stress. A few embryos were transferred to M16 medium (isotonic) and were cultured till they progressed to the blastocyst stage.

### Exposure of NFE and HPE to Oxidative Stress

Hydrogen peroxide (Cat. No. 1.93407.0521, Merck) was freshly added to sterile M16 medium. NFE and HPE were exposed at 2-cell stage to 25 µM of H_2_O_2_ for 30 min at 37 °C. Embryos were washed 3 times in M16 medium and used for further experiments. The control group embryos were cultured in M16 medium. Intracellular reactive oxygen species, MMP, and ER stress levels were assessed in 2-cell stage embryos, while to understand the developmental potential, embryos were cultured in M16 media till the blastocyst stage.

### Immunofluorescence

Two-cell stage embryos were fixed in 4% paraformaldehyde overnight at 4 °C followed by wash and permeabilization using 0.5% Triton X-100 (Cat. No. 2024271, Sisco Research Laboratories, India) for 15 min. The embryos were then kept in blocking solution (10% goat serum, Cat. No. X0907, DAKO, Denmark) for 1 h at room temperature, followed by incubating overnight with appropriate dilutions of primary antibodies [1:300 anti-GRP78 (Cat. No. SAB4501452, Sigma, USA), and 1:200 anti-XBP-1 antibody (Cat. No. ab37152, Abcam, USA), diluted in blocking solution] at 4 °C. Embryos were washed and incubated with secondary antibody (1:500 and 1:300 dilution of goat anti-rabbit IgG Alexa Fluor 488, Cat. No. ab150077, Abcam, USA for GRP78 and XBP-1, respectively), for 2 h at room temperature. The nuclei were stained with 4,6-diamidino-2-phenylindole (DAPI) (Cat. No. D9542, Sigma-Aldrich, USA), and fluorescence images were acquired using a fluorescence microscope (Axio Imager A1, Carl Zeiss, Gottingen, Germany), and the fluorescence intensity was quantified using Q-Capture software (Q-Capture Pro 7, USA) [32].

### Mitochondrial Membrane Potential by JC-1 Staining

Mitochondrial membrane potential in 2-cell stage embryos was determined as described by Reers et al. [38]. Briefly, the embryos were incubated in culture media containing 10 µg/mL of JC-1 (5,5′,6,6′-tetrachloro-1,1′,3,3′-tetraethylbenzimidazolylcarbocyanine iodide, Cat. No. T3168, Molecular probes, Life technologies, USA) for 30 min at 37 °C, followed by a wash in M16 medium. The JC-1 monomers and JC-1 aggregates were assessed using a fluorescence microscope. The mitochondrial potential was calculated using ImageJ software (National Institute of Health, Bethesda, MD, USA).

### Assessment of ROS Level

The intracellular ROS level in the embryos was assessed using the dichlorodihydrofluorescein diacetate (DCFH-DA, Cat. No. D6883, Sigma-Aldrich, USA) assay as described by Kalthur et al. [39]. In brief, the 2-cell stage embryos were incubated for 30 min in 10 mM DCFH-DA in M16 media droplets (prewarmed) which were maintained at 37 °C temperature and 5% CO_2_. Embryos were then washed 3–4 times in M16 medium and mounted on a clean glass slide using mounting medium (Cat. No. S3023, Dako, Carpinteria, CA, USA). The embryos were observed and imaged under a fluorescence microscope at 400 × magnification. Fluorescence intensity was estimated using Q-Capture software.

### Detection of DNA Damage in Blastocyst by Terminal Deoxynucleotidyl Transferase dUTP Nick-End Labeling (TUNEL) Assay

Blastocysts were washed in phosphate-buffered saline (PBS) with 0.5% BSA to remove traces of culture medium and fixed in 4% paraformaldehyde overnight at 4 °C. The embryos were washed 3 times in PBS with 0.5% BSA followed by permeabilization in 0.1% sodium citrate and 0.5% Triton X 100 in PBS with 0.5% BSA for 1 h at room temperature. Later, the embryos were washed 3 times in PBS with 0.5% BSA and incubated with TUNEL reaction mixture at 37 °C in a humidified chamber in the dark for 1 h. The embryos were washed 3 times in PBS with 0.5% BSA, counterstained with DAPI, and mounted on a glass slide. The blastocysts were scored under fluorescence microscope, and the TUNEL index was calculated by dividing the total number of TUNEL positive cells by total cell number in the embryo (DAPI positive cells) [39].

### Statistical Analysis

All values (except blastocyst rate expressed in percentage) are expressed as mean ± SE. Statistical analysis was carried out using the GraphPad Prism 8.0.1 software, CA, USA. The differences in blastocyst rate were analyzed by chi-square test, while for other parameters, the two-way analysis of variance (ANOVA)–Tukey’s multiple comparison test or Sidak’s multiple comparison test were used to compare various groups. The significance level *p* < 0.05 was considered as statistically significant. The significance for intra-group comparisons is denoted as **** for *p* < 0.0001, *** for *p* < 0.001, ** for *p* < 0.01, and * for *p* < 0.05, and for inter-group comparisons, it is denoted as a for *p* < 0.0001, b for *p* < 0.001, c for *p* < 0.01, and d for *p* < 0.05.

## Results

### ER Stress Response Activation in 2-Cell Stage of NFE and HPE to Environmental Stressors

The expression of ER stress markers GRP78 and XBP-1 were used to understand the ER stress response in the embryos upon treatment with various stress conditions. The GRP78 is a centrally located, monomeric, globular protein that modulates the UPR coping response by functionally sorting and releasing the terminally misfolded substrates to the ER-associated degradation (ERAD) pathway [23]. A subset of genes activated during the ER stress-induced UPR is in turn regulated by the transcription factor XBP-1 [23]. In our study, both GRP78 and XBP-1 proteins were found to be localized in the cytoplasm of 2-cell stage embryos.

### GRP78 Expression

**Fig. 2:**
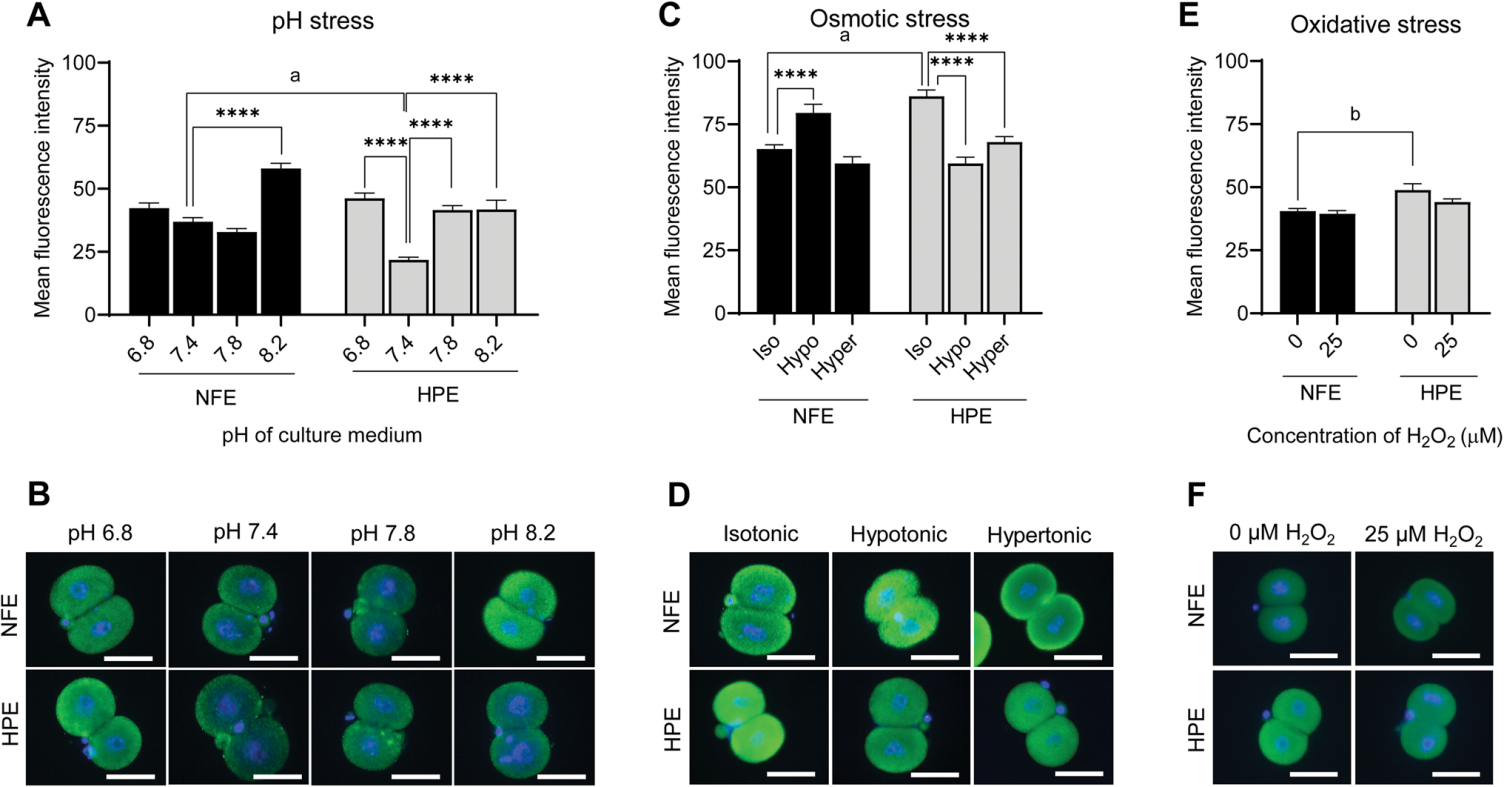
Effect of various stress in NFE and HPE on the GRP78 expression in 2-cell stage embryos in vitro. **A** GRP78 expression in 2-cell stage embryos exposed to pH stress. **B** Representative images of GRP78 expression pattern in 2-cell stage embryos exposed to pH stress studied by immunofluorescence (400 ×). **C** GRP78 expression in 2-cell stage embryos exposed to osmotic stress. **D** Representative images of GRP78 expression pattern in 2-cell stage embryos exposed to osmotic stress studied by immunofluorescence (400 ×). **E** GRP78 expression in 2-cell stage embryos exposed to oxidative stress. **F** Representative images of GRP78 expression pattern in 2-cell stage embryos exposed to oxidative stress studied by immunofluorescence (400 ×). Scale bar = 50 µM; **** *p* < 0.0001; a, *p* < 0.0001; b, *p* < 0.001. The number of embryos per group (*n*): pH stress, NFE pH 6.8 (*n* = 20), NFE pH 7.4 (*n* = 29), NFE pH 7.8 (*n* = 22), NFE pH 8.2 (*n* = 23), HPE pH 6.8 (*n* = 34), HPE pH 7.4 (*n* = 39), HPE pH 7.8 (*n* = 38), and HPE pH 8.2 (*n* = 33); osmotic stress, NFE isotonic (*n* = 56), NFE hypotonic (*n* = 53), NFE hypertonic (*n* = 52), HPE isotonic (*n* = 43), HPE hypotonic (*n* = 43), and HPE hypertonic (*n* = 40); and oxidative stress, NFE 0 µM H_2_O_2_ (*n* = 69), NFE 25 µM H_2_O_2_ (*n* = 68), HPE 0 µM H_2_O_2_ (*n* = 61), and HPE 25 µM H_2_O_2_ (*n* = 59)

Culturing NFE in acidic pH (6.8) and moderate alkaline pH (7.8) did not alter the GRP78 expression (42.30 ± 2.10 and 32.85 ± 1.37, respectively) compared to those cultured in pH 7.4 (36.97 ± 1.53). However, when cultured in the extreme alkaline pH (8.2), the NFE showed significantly higher GRP78 levels (58.0 ± 2.08, *p* < 0.0001). On the other hand, the HPE showed a two-fold higher GRP78 expression when cultured with the medium of pH 6.8, 7.8, and 8.2 (46.21 ± 2.06, 41.57 ± 1.72, and 41.89 ± 3.65, respectively) compared to the embryos cultured in media with pH 7.4 (21.75 ± 1.14, *p* < 0.0001) (Fig. 2A and B), indicating HPE are more susceptible to pH stress.

When the embryos were exposed to osmotic stress, the NFE showed a significantly higher expression of GRP78 in the hypotonic media (79.58 ± 3.36) compared to the isotonic media (65.16 ± 1.68, *p* < 0.0001) (Fig. 2C and D). Exposure to hypertonic media did not have any influence on the GRP78 expression (59.42 ± 2.65). On the contrary, significantly lower GRP78 expression was observed in HPE, when exposed to both hypotonic (59.45 ± 2.51) and hypertonic conditions (67.92 ± 2.19) compared to isotonic conditions (86.12 ± 2.51, *p* < 0.0001).

When we studied the GRP78 expression pattern following exposure to oxidative stress (25 µM H_2_O_2_), NFE did not show any significant change in the expression pattern (Fig. 2E and F). In HPE, the GRP78 level was non-significantly lower (44.11 ± 1.28) following exposure to oxidative stress, compared to the unexposed HPE (48.84 ± 2.48).

### XBP-1 Expression

**Fig. 3:**
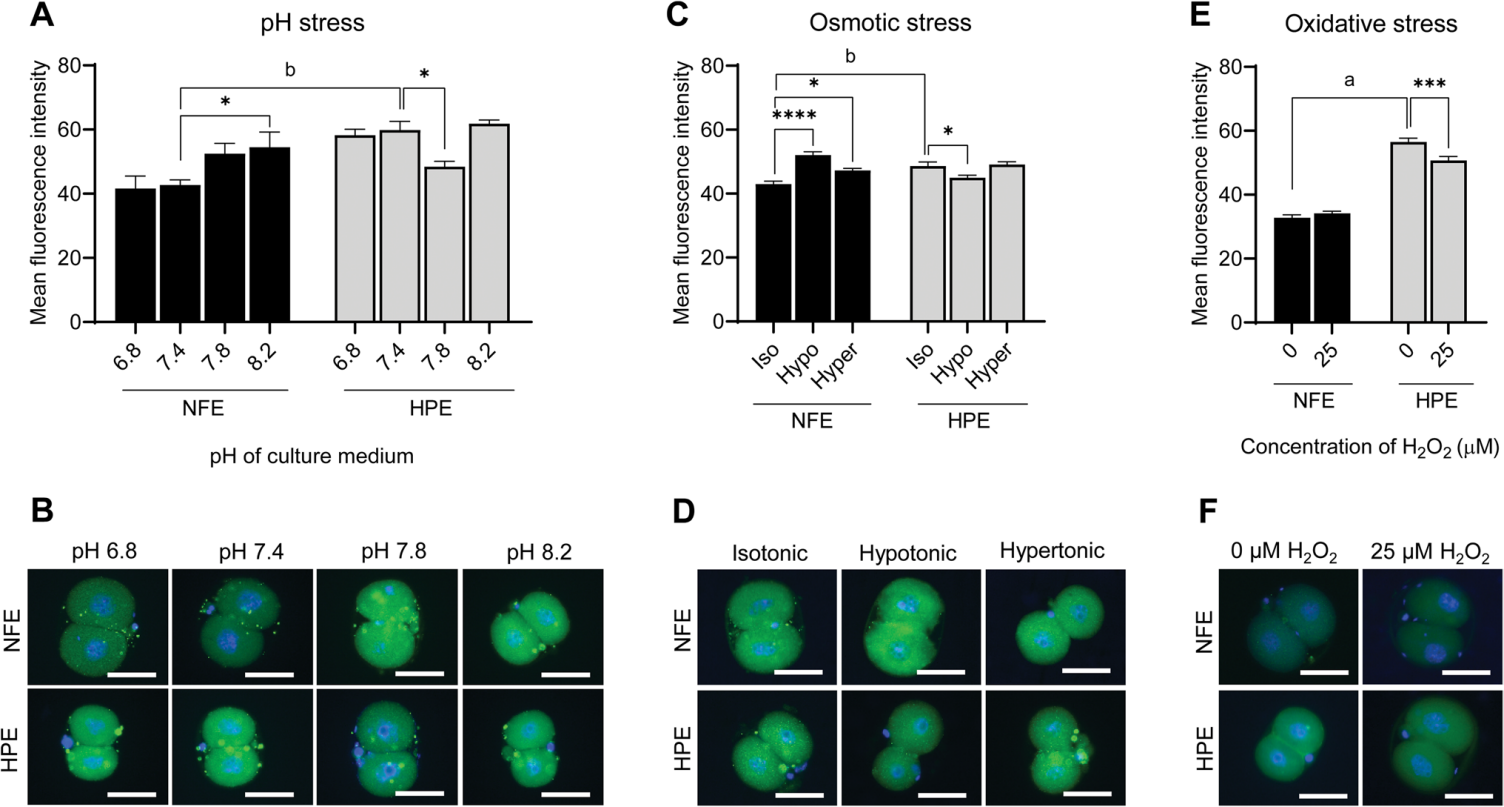
Effect of various stress in NFE and HPE on the XBP-1 expression in 2-cell stage embryos in vitro. **A** XBP-1 expression in 2-cell stage embryos exposed to pH stress. **B** Representative images of XBP-1 expression pattern in 2-cell stage embryos exposed to pH stress studied by immunofluorescence (400 ×). **C** XBP-1 expression in 2-cell stage embryos exposed to osmotic stress. **D** Representative images of XBP-1 expression pattern in 2-cell stage embryos exposed to osmotic stress studied by immunofluorescence (400 ×). **E** XBP-1 expression in 2-cell stage embryos exposed to oxidative stress. **F** Representative images of XBP-1 expression pattern in 2-cell stage embryos exposed to oxidative stress studied by immunofluorescence (400 ×). Scale bar = 50 µM; * *p* < 0.05; *** *p* < 0.001; **** *p* < 0.0001; a, *p* < 0.0001; b, *p* < 0.001. The number of embryos per group (*n*): pH stress, NFE pH 6.8 (*n* = 25), NFE pH 7.4 (*n* = 25), NFE pH 7.8 (*n* = 23), NFE pH 8.2 (*n* = 23), HPE pH 6.8 (*n* = 21), HPE pH 7.4 (*n* = 24), HPE pH 7.8 (*n* = 24), and HPE pH 8.2 (*n* = 23); osmotic stress, NFE isotonic (*n* = 15), NFE hypotonic (*n* = 14), NFE hypertonic (*n* = 15), HPE isotonic (*n* = 18), HPE hypotonic (*n* = 20), and HPE hypertonic (*n* = 20); and oxidative stress, NFE 0 µM H_2_O_2_ (*n* = 39), NFE 25 µM H_2_O_2_ (*n* = 41), HPE 0 µM H_2_O_2_ (*n* = 74), and HPE 25 µM H_2_O_2_ (*n* = 73)

XBP-1 expression was found to be significantly higher in HPE, when compared to NFE at the 2-cell stage (*p* < 0.001) (Fig. 3A). The exposure of NFE to acidic pH did not cause any significant effect on XBP-1 expression (41.64 ± 3.85), while under alkaline condition (pH 7.8), there was a non-significant increase in expression (52.51 ± 3.19), and under extreme alkaline condition (pH 8.2), there was a significant increase (54.50 ± 4.72, *p* < 0.05), compared to those exposed to pH 7.4 (42.72 ± 1.55) (Fig. 3A and B). However, in HPE, exposure to either acidic or alkaline pH did not alter XBP-1 expression compared to control, except at pH 7.8, in which there was a significant decrease in the expression level (*p* < 0.05).

The NFE exposed to hypotonic (52.02 ± 1.10) and hypertonic stress (47.26 ± 0.66) showed significantly higher expression of XBP-1 compared to isotonic exposure (42.99 ± 0.88) (Fig. 3C and D). The HPE on the other hand showed significantly lower XBP-1 levels (*p* < 0.05) when exposed to hypotonic stress (44.97 ± 0.78) compared to isotonic exposure (48.62 ± 1.29), while the hypertonic stress exposure did not alter the XBP-1 expression (49.06 ± 0.88). The NFE exposed to oxidative stress (Fig. 3E and F) did not show any changes in XBP-1 levels, while the HPE exposed to oxidative stress showed significantly lower XBP-1 levels compared to control (*p* < 0.001).

### Mitochondrial Membrane Potential (MMP) in NFE and HPE at 2-Cell Stage After Exposure to Environmental Stressors

**Fig. 4:**
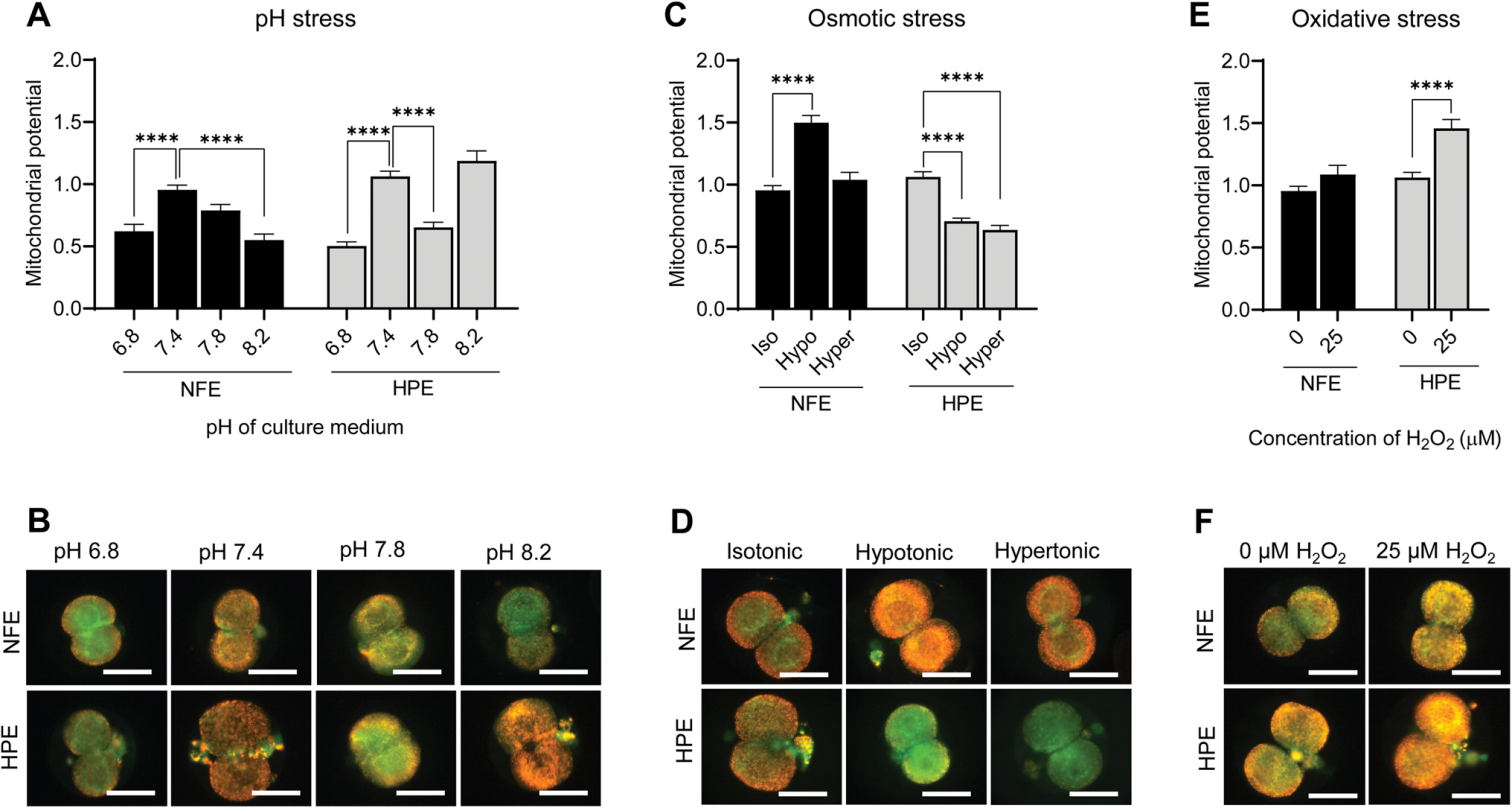
**A** Mitochondrial membrane potential in NFE and HPE after pH stress exposure in 2-cell stage embryos. **B** Representative images of NFE and HPE exposed to pH stress stained with JC-1 (400 ×). **C** Mitochondrial membrane potential in NFE and HPE after osmotic stress exposure in 2-cell stage embryos. **D** Representative images of NFE and HPE exposed to osmotic stress stained with JC-1 (400 ×). **E** Mitochondrial membrane potential in NFE and HPE after oxidative stress exposure in 2-cell stage embryos. **F** Representative images of NFE and HPE exposed to oxidative stress stained with JC-1 (400 ×). Scale bar = 50 µM; **** *p* < 0.0001. The number of embryos per group (*n*): pH stress, NFE pH 6.8 (*n* = 47), NFE pH 7.4 (*n* = 201), NFE pH 7.8 (*n* = 52), NFE pH 8.2 (*n* = 48), HPE pH 6.8 (*n* = 34), HPE pH 7.4 (*n* = 154), HPE pH 7.8 (*n* = 33), and HPE pH 8.2 (*n* = 35); osmotic stress, NFE isotonic (*n* = 201), NFE hypotonic (*n* = 45), NFE hypertonic (*n* = 64), HPE isotonic (*n* = 154), HPE hypotonic (*n* = 78), and HPE hypertonic (*n* = 76); and oxidative stress, NFE 0 µM H_2_O_2_ (*n* = 201), NFE 25 µM H_2_O_2_ (*n* = 87), HPE 0 µM H_2_O_2_ (*n* = 154), and HPE 25 µM H_2_O_2_ (*n* = 47)

No significant changes were observed in MMP of NFE and HPE at 2-cell stage (Fig. 4A). Exposure of NFE to both acidic and alkaline pH resulted in reduced MMP (0.62 ± 0.05, 0.78 ± 0.04, and 0.55 ± 0.04 in pH 6.8, 7.8, and 8.2, respectively) when compared to the embryos exposed to media with pH 7.4 (0.95 ± 0.03). However, the difference was significant only for embryos exposed to pH 6.8 and 8.2 (*p* < 0.0001) (Fig. 4A and B). Exposure of HPE to pH 6.8 (0.50 ± 0.03) leads to significantly lower MMP (*p* < 0.0001) compared to those cultured at pH 7.4 (1.06 ± 0.04). When the HPE were exposed to media with alkaline pH 7.8, the expression decreased significantly (0.65 ± 0.04, *p* < 0.0001), while at pH 8.2, the embryos had non-significantly higher MMP (1.18 ± 0.07) in comparison to those exposed to media with pH 7.4.

Exposure of NFE to hypotonic conditions resulted in significantly higher MMP (1.49 ± 0.05, *p* < 0.0001) compared to those cultured in isotonic conditions (0.95 ± 0.03) (Fig. 4C and D), while exposure to hypertonic condition did not affect the MMP (1.03 ± 0.06). On the other hand, the MMP was significantly lower (*p* < 0.0001) in HPE upon hypotonic (0.70 ± 0.02) as well as hypertonic exposure (0.63 ± 0.03) compared to the isotonic conditions (1.06 ± 0.04).

Exposure to 25 µM H_2_O_2_ did not affect the MMP in the NFE (1.08 ± 0.07). However, in the HPE exposed to oxidative stress, MMP was significantly higher (1.45 ± 0.07, *p* < 0.0001) (Fig. 4E and F).

### Alterations in Intracellular ROS in NFE and HPE at 2-Cell Stage After Exposure to Exogenous H_2_O_2_

**Fig. 5:**
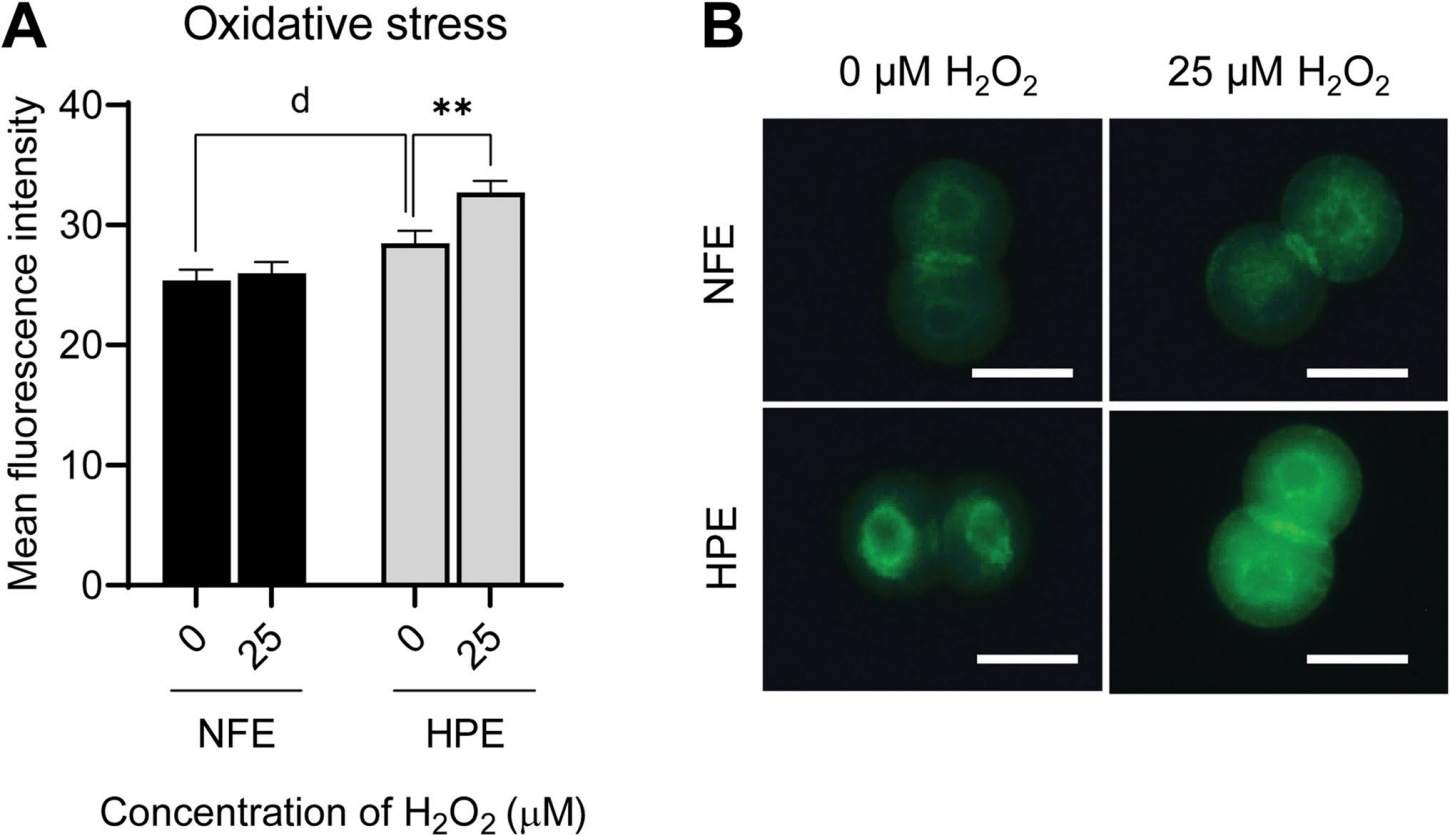
**A** Intracellular reactive oxygen species (ROS) level in NFE and HPE exposed to oxidative stress at 2-cell stage assessed by staining. **B** Representative images of 2-cell stage embryos exposed to oxidative stress stained with DCFH-DA dye (400 ×). Scale bar = 50 µM; ** *p* < 0.01; d, *p* < 0.05. The number of embryos per group (*n*): oxidative stress, NFE 0 µM H_2_O_2_ (*n* = 25), NFE 25 µM H_2_O_2_ (*n* = 21), HPE 0 µM H_2_O_2_ (*n* = 26), and HPE 25 µM H_2_O_2_ (*n* = 19)

The basal level of intracellular ROS was significantly higher in HPE when compared to NFE (25.39 ± 0.87 and 28.48 ± 1.04 in NFE and HPE, respectively, *p* < 0.05) (Fig. 5A and B). The intracellular ROS levels were assessed in 2-cell stage embryos following 30 min exposure to oxidative stress. Exposure to 25 µM of H_2_O_2_ in NFE showed no change (25.97 ± 0.95), while in the HPE, the ROS level was observed to be significantly elevated (32.71 ± 0.97, *p* < 0.01) compared to the control embryos.

### Impact of Environmental Stressors on Blastocyst Development of NFE and HPE

**Fig. 6:**
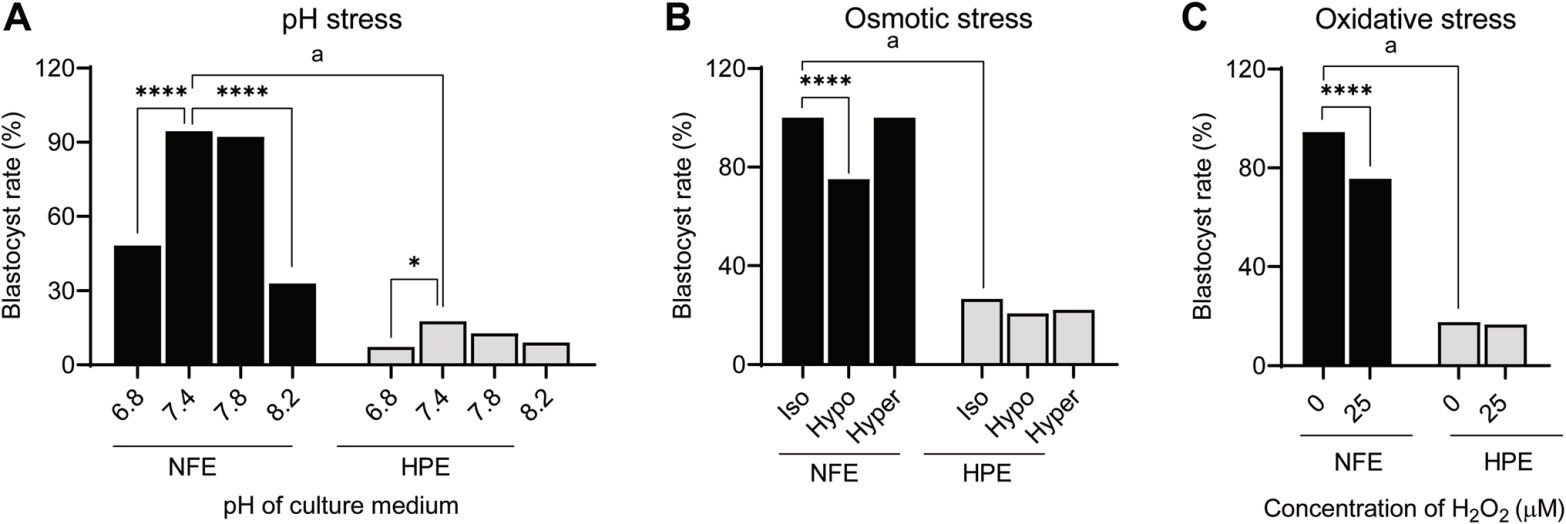
Blastocyst rate in NFE and HPE exposed to **A** pH stress; **B** osmotic stress; and **C** oxidative stress at 2-cell stage. * *p* < 0.05; **** *p* < 0.0001; a, *p* < 0.0001. The number of embryos per group (*n*): pH stress, NFE pH 6.8 (*n* = 20), NFE pH 7.4 (*n* = 29), NFE pH 7.8 (*n* = 22), NFE pH 8.2 (*n* = 23), HPE pH 6.8 (*n* = 34), HPE pH 7.4 (*n* = 39), HPE pH 7.8 (*n* = 38), and HPE pH 8.2 (*n* = 33); osmotic stress, NFE isotonic (*n* = 56), NFE hypotonic (*n* = 53), NFE hypertonic (*n* = 52), HPE isotonic (*n* = 43), HPE hypotonic (*n* = 43), and HPE hypertonic (*n* = 40); and oxidative stress, NFE 0 µM H_2_O_2_ (*n* = 69), NFE 25 µM H_2_O_2_ (*n* = 68), HPE 0 µM H_2_O_2_ (*n* = 61), and HPE 25 µM H_2_O_2_ (*n* = 59)

HPE exhibited a significantly lower blastocyst rate (17.64%, *p* < 0.0001) compared to NFE (94.44%) when cultured in M16 media with pH 7.4 (Fig. 6A). Blastocyst rate significantly reduced (*p* < 0.0001) when the NFE were exposed to acidic (pH 6.8, 48.18%) and extreme alkaline condition (pH 8.2, 32.95%). Similarly, exposure of HPE to either acidic (pH 6.8) or alkaline pH conditions (pH 7.8 or 8.2) resulted in a decrease in the blastocyst rate compared to the control. However, the reduction in blastocyst rate was significant only when the 2-cell stage embryos were exposed to pH 6.8 (7.29%, *p* < 0.05).

Subjecting the NFE to hypotonic stress affected the developmental potential, as evident from a significant decrease in the blastocyst rate (100% vs 75% in isotonic and hypotonic conditions respectively, *p* < 0.0001) (Fig. 6B). However, they exhibited tolerance towards hypertonic stress as the blastocyst rate in these embryos was not affected (100%). The HPE on the other hand were susceptible to both hypertonic and hypotonic stress with a non-significant decrease in blastocyst rate (26.64%, 20.75%, and 22.22% in isotonic, hypotonic, and hypertonic conditions, respectively).

NFE exposed to oxidative stress exhibited significantly lower blastocyst rate (94.44% v/s 75.58% in 0 and 25 µM H_2_O_2_ respectively, *p* < 0.0001) (Fig. 6C). However, exposure to oxidative stress did not have any significant detrimental effect on the developmental potential of the HPE (17.64 and 16.66% in 0 and 25 µM H_2_O_2_, respectively).

### Effect of Environmental Stressors on Blastocyst Fitness of NFE and HPE

**Fig. 7:**
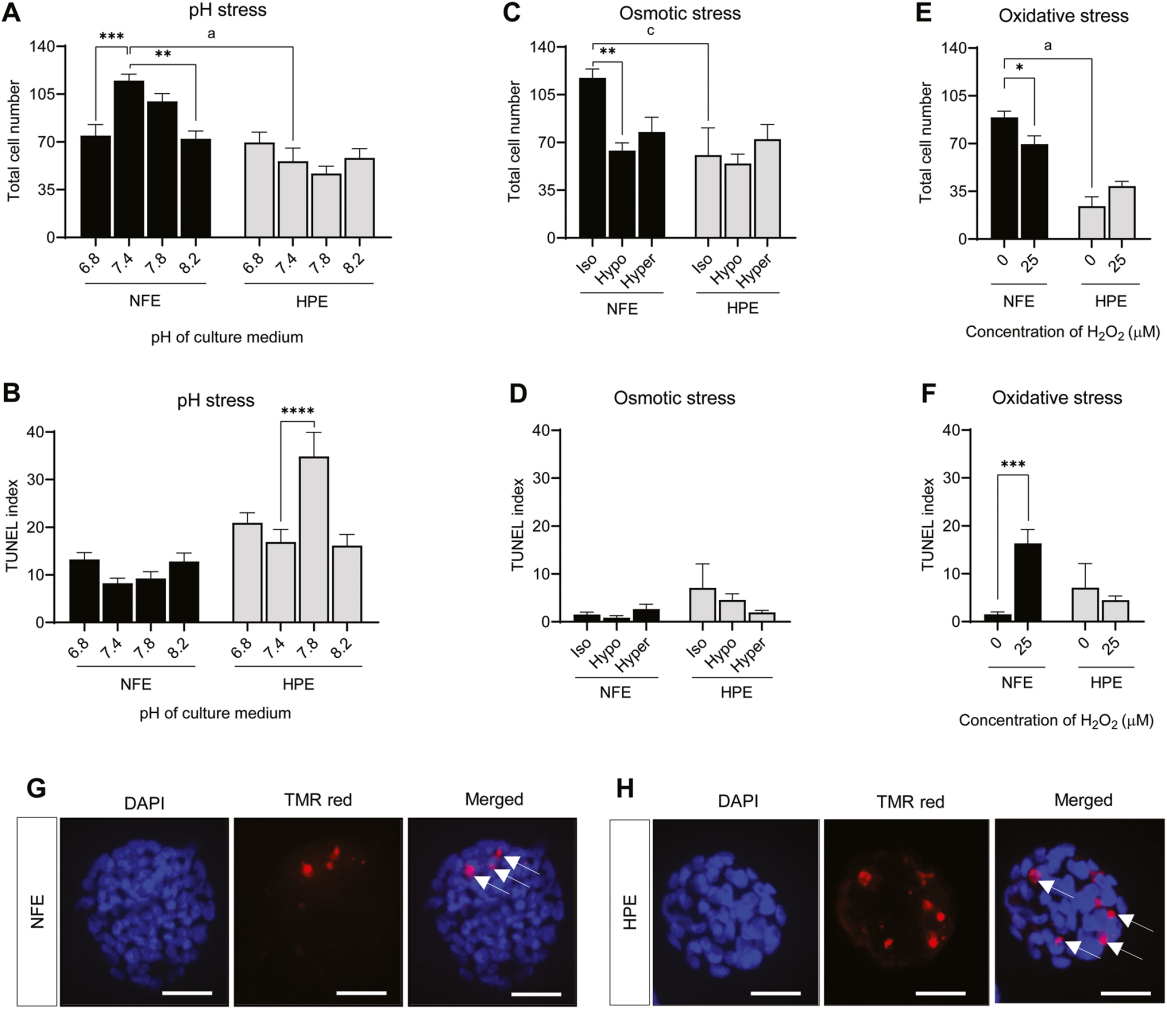
Total cell number in blastocysts derived from NFE and HPE exposed to **A** pH stress; **C** Osmotic stress; and **E** oxidative stress at 2-cell stage. TUNEL index in blastocysts derived from NFE and HPE exposed to **B** pH stress; **D** osmotic stress; and **F** oxidative stress at 2-cell stage. Representative images of blastocysts stained with DAPI for total cell number count (400 ×) and TUNEL index in blastocysts of **G** NFE and **H** HPE (400 ×). The white arrows indicate the apoptotic cells in the blastocyst. Scale bar = 50 µM; * *p* < 0.05; ** *p* < 0.01; *** *p* < 0.001; **** *p* < 0.0001; a, *p* < 0.0001; c, *p* < 0.01. The number of embryos per group (*n*): pH stress, NFE pH 6.8 (*n* = 24), NFE pH 7.4 (*n* = 25), NFE pH 7.8 (*n* = 16), NFE pH 8.2 (*n* = 15), HPE pH 6.8 (*n* = 15), HPE pH 7.4 (*n* = 8), HPE pH 7.8 (*n* = 12), and HPE pH 8.2 (*n* = 15); osmotic stress, NFE isotonic (*n* = 10), NFE hypotonic (*n* = 17), NFE hypertonic (*n* = 8), HPE isotonic (*n* = 12), HPE hypotonic (*n* = 14), and HPE hypertonic (*n* = 14); and oxidative stress, NFE 0 µM H_2_O_2_ (*n* = 10), NFE 25 µM H_2_O_2_ (*n* = 19), HPE 0 µM H_2_O_2_ (*n* = 10), and HPE 25 µM H_2_O_2_ (*n* = 28)

The blastocysts obtained after exposure of 2-cell embryos to pH stress, osmotic stress, and oxidative stress were analyzed for blastocyst fitness by counting the total cell number and DNA damage foci (Fig. 7G and H). The blastocysts obtained from HPE were observed to be of poor quality when compared to the NFE, with significantly lower cell number (*p* < 0.0001) and higher TUNEL index (Fig. 7A–F). Blastocysts derived from NFE exposed to acidic and alkaline pH had lower total cell number (74.62 ± 7.97 (*p* < 0.001), 99.68 ± 5.77 and 72.26 ± 5.73 (*p* < 0.01) in pH 6.8, 7.8, and 8.2 respectively) (Fig. 7A) compared to those cultured in pH 7.4 (114.88 ± 4.77). In addition, the apoptotic index was higher in blastocysts of these groups compared to control (13.69 ± 2.02, 8.63 ± 1.47, 9.24 ± 1.45, and 13.68 ± 2.11 in NFE exposed to pH 6.8, 7.4, 7.8, and 8.2 pH, respectively) (Fig. 7B). On the other hand, the parthenogenetic embryos showed no significant difference in total cell number of blastocysts derived from 2-cell stage embryos exposed to acidic pH (pH 6.8, 69.60 ± 7.59) or extreme alkaline pH (pH 8.2, 58.33 ± 6.66), compared to the control group blastocysts (pH 7.4, 55.87 ± 9.62). However, the blastocysts from HPE cultured in media with pH 7.8 showed a lower total cell number (46.83 ± 5.32). The DNA integrity of blastocysts from HPE was unaffected when cultured in the acidic (20.93 ± 2.08) and extreme alkaline pH (16.15 ± 2.38), compared to control (16.90 ± 2.66). But the blastocysts from embryos exposed to pH 7.8 showed a significantly higher level of DNA damage (34.84 ± 5.12, *p* < 0.0001).

The total cell number in blastocysts obtained from NFE exposed to hypotonic and hypertonic condition was half of the NFE cultured in isotonic conditions (117.27 ± 06.60, 63.94 ± 05.78, and 71.00 ± 11.67 in control, hypotonic, and hypertonic conditions respectively) (Fig. 7C), with significantly lower cell number in hypotonic condition (*p* < 0.01). However, the cell number in HPE was unaffected after hypotonic and hypertonic exposure at the 2-cell stage. Though the basal level of apoptosis was already high in parthenotes (7.07 ± 5.05) compared to the normally fertilized embryos (1.52 ± 0.49) (Fig. 7D), it was seen to be lower in both types of embryos upon hypotonic stress exposure, while it showed a differential response in hypertonic conditions. The NFE exposed to hypertonic stress that successfully reached the blastocyst stage seemed to have higher apoptotic index than those that survived hypotonic stress (0.85 ± 0.42 and 2.35 ± 0.95 in hypotonic and hypertonic conditions, respectively). The blastocysts from HPE on the other hand showed lower apoptosis in both hypotonic and hypertonic stress conditions.

The blastocysts from NFE exposed to oxidative stress had significantly lower cell number (69.58 ± 06.09, *p* < 0.05) (Fig. 7E) and higher apoptosis (16.37 ± 02.88, *p* < 0.001) (Fig. 7F) compared to unexposed control (89.18 ± 04.61, and 01.52 ± 0.49 total cell number and TUNEL index respectively). However, in blastocysts from HPE exposed to oxidative stress, total cell number was marginally higher (38.75 ± 3.54) compared to that of the control (24.10 ± 06.93). The apoptotic index in the exposed group was also marginally lower, showing an increased resistance in HPE to oxidative stress-induced DNA damage when compared to NFE.

## Discussion

In this study, we have demonstrated the differential response of the NFE and the HPE to environmental stressors associated with in vitro culture. Further, we observed that the tolerance of embryos at the preimplantation stage varied for different types of stressors. Based on our findings, the absence of paternal factors made HPE more susceptible to environmental stressors, which suggests that the paternal factors may have a significant contribution to stress tolerance during earliest stages of embryo development.

The preimplantation-stage embryos are known to get exposed to various stress during their in vitro culture, since the culture conditions only partially mimic their natural environment in the female reproductive tract [40]. The embryos exposed to exogenous stress have a reduced fitness [41], leading to poor developmental and fetal anomalies [40]. Moreover, impaired sperm quality is found to be associated with delayed fertilization and poor embryonic morphology [42, 43].

Preimplantation embryos in the female reproductive tract are in motion and exposed to changing hormones, nutrients, growth factors, cytokines, and a varied range of pH, with a markedly alkaline environment in the oviduct and a more acidic uterine environment [1, 44, 45]. The internal pH of the mammalian embryos in vivo is maintained in the range between 7.1 and 7.2, using two major mechanisms: the Na + /H + antiporter, which regulates acid loads, and the HCO_3_^–^/Cl^–^ anion exchanger (AE), which regulates the alkaline load [46, 47]. The alkalosis defense mechanisms (AE-2 and AE-4) are known to be functional in the mouse preimplantation-stage embryos as early as the pronuclear (PN) stage [5].

During in vitro culture, the embryos are exposed to a static environment with limited nutrients, where they come in contact with end products of metabolism, which alter the pH of the culture media [1]. These changes in the external pH can in turn result in alterations in the internal pH of early embryos [48]. A study by Zander-Fox et al. [49] demonstrated that preimplantation embryos are highly sensitive to a small decrease in internal pH, and either short-term or extended exposure to reduced internal pH significantly affected the developing embryos.

Nematollahi-mahani et al. [50] have reported that the mouse embryos at 2-cell stage are tolerant around pH 7.0–7.6. In line with this, in our study, we observed that the NFE are sensitive to acidic and extreme alkaline pH (pH 6.8 and 8.2) indicated by the poor MMP, reduced blastocyst rate, lower total cell number, and increased apoptosis at the blastocyst stage. However, at pH 7.8, the development of NFE was similar to the control (pH 7.4). The haploid parthenotes on the other hand were found to be sensitive to any pH fluctuations (pH 6.8, 7.8, and 8.2) shown by increased GRP78 levels and aberrant MMP at the 2-cell stage and reduced blastocyst rate with lower total cell number and increased apoptosis in the blastocysts. The NFE were seen to have a broader range of pH tolerance compared to HPE.

Osmolarity is one of the physical factors affecting the development of preimplantation-stage embryos. The osmolarity found in the mouse oviduct ranges from 290 to 300 mOsmol/L [51], while the osmolarity of the commercially available in vitro embryo culture media is generally around 260 mOsmol/L [52]. Dawson and Baltz [53] have reported that in vitro exposure to hyperosmolarity affects the preimplantation development in normally fertilized mice embryos. But our results stand contrary to these findings, as we saw the blastocyst rate in the NFE exposed to the hypertonic solution was similar to the embryos cultured in isotonic conditions. However, it is important to note that Dawson and Baltz [53] assessed these responses using in vivo derived embryos and used NaCl to generate the hyperosmotic conditions. On the other hand, we used 0.01 M sucrose (390 mOsmol/L) to test the effect of hyperosmotic conditions and IVF-derived embryos.

Blastocysts obtained from the hypertonic exposed NFE had a decreased total cell number and increased apoptosis. The NFE showed sensitivity to hypotonic stress (191 mOsmol/L), indicated by the poor blastocyst rate with decreased total cell number. The early responses included increased GRP78 levels and aberrant MMP. The HPE were found to be sensitive to both hypotonic as well as hypertonic stress with decreased GRP78, XBP-1 levels, and MMP in the early stages, ultimately reducing the blastocyst rate.

The preimplantation embryos are known to regulate their redox environment for optimal development both in vivo and in vitro. ROS are generated as a result of cellular metabolism and act like a double-edged sword. Physiological levels of ROS work as helpful signaling molecules that are necessary to maintain normal embryo development. When present in excess, the ROS may prove to be deleterious to embryos and impair normal development [54].

Various studies have used H_2_O_2_ exposure in vitro to study the deleterious effects of ROS on preimplantation embryo development. In a study by Cebral et al. [55], 25 µM H_2_O_2_ inhibited the blastocyst formation in mouse embryos when exposed at the 2-cell stage. Qian et al. [56] reported that the preimplantation embryos exposed to 30 µM H_2_O_2_ for 30 min showed decreased blastocyst rates and increased apoptosis, which was consistent with our results where we used 25 µM H_2_O_2_ for 30 min.

The early-stage embryo response to oxidative stress showed increased ROS levels, mitochondrial damage [57], and decreased MMP [56]. Contrary to this, the normally fertilized embryos in this study did not show any difference in ROS and MMP levels. In addition to this, the ER stress response markers GRP78 and XBP-1 were also unaltered. This difference is possibly due to the difference in the stage of exposure of H_2_O_2_ as Qian et al. [56] did the H_2_O_2_ treatment at 1-cell stage, while in our study, the treatment was done at the 2-cell stage, which is also the stage of embryo genome activation in mice embryos.

In our study, HPE showed a differential response upon exposure to oxidative stress, when compared to NFE. The early response showed an increase in ROS levels, a decrease in ER stress response protein (XBP-1), and an abnormal increase in MMP. The aberrant shift in the MMP of preimplantation embryos is associated with decreased developmental potential [17]. In the present study, despite the early responses, there was no change observed in the blastocyst rates of HPE exposed to oxidative stress, when compared to the untreated HPE. Further, based on the ability of the embryos to progress to the blastocyst stage and the quality of the blastocysts developed, it is clearly evident that early and late response in NFE and HPE differ following their exposure to various in vitro stress.

When compared to the NFE, the HPE had compromised developmental potential characterized by poor blastocyst rate and lower total cell number even when cultured under optimal conditions, indicating poor proliferation, which are in line with earlier reports [32, 58–60]. It has been reported that haploidy can lead to high incidence of apoptosis when compared to diploid parthenogenetic embryos and NFE [61], which is consistent with our findings (in the case of NFE and HPE). However, the limitation of our study is that we did not use diploid parthenogenetic embryos in addition to HPE, which would have helped us to rule out the contribution of the ploidy status of embryos in their stress tolerance. Nonetheless, the studies have used haploid parthenotes as a model to understand the paternal contribution to early embryo development [32, 60].

In conclusion, this study provides experimental evidence on the unique sensitivity of haploid parthenogenetic embryos to in vitro stress conditions when compared to normally fertilized embryos (Table 1), which points towards the significance of the contribution of paternal factors and/or ploidy status of preimplantation embryos to the stress response during early embryogenesis.

**Table 1:** Stress response in NFE and HPE exposed to pH, osmotic and oxidative stress at 2-cell stage embryos of Swiss albino mice

Stress type	Parameters	NFE	HPE
pH stress	GRP78	↑ in pH 8.2	↑ in pH 6.8, 7.8 and 8.2
	XBP-1	↑ in pH 8.2	↓ in pH 7.8
	Mitochondrial potential	↓ in pH 6.8 and 8.2	↓ in pH 6.8, 7.8; ↑ in pH 8.2
	Blastocyst rate	↓ in pH 6.8 and 8.2	↓ in pH 6.8, 7.8 and 8.2
	Total cell number	↓ in pH 6.8 and 8.2	↓ in pH 7.8
	TUNEL index	↑ in pH 6.8 and 8.2	↑ in pH 6.8 and 7.8
Osmotic stress	GRP78	↑ in hypo	↓ in hypo and hyper
	XBP-1	↑ in hypo and hyper	↓ in hypo
	Mitochondrial potential	↑ in hypo	↓ in hypo and hyper
	Blastocyst rate	↓ in hypo	↓ in hypo and hyper
	Total cell number	↓ in hypo, hyper	No change
	TUNEL index	↓ in hypo; ↑ in hyper	↓ in hypo and hyper
Oxidative stress	ROS	No change	↑
	GRP78	No change	No change
	XBP-1	No change	↓
	Mitochondrial potential	No change	↑
	Blastocyst rate	↓	No change
	Total cell number	↓	↑
	TUNEL index	↑	No change

## Data Availability

The authors declare that all the data supporting the findings of this study are available within the article.
